# Prevention of experimental autoimmune myasthenia gravis by rat Crry-Ig: A model agent for long-term complement inhibition in vivo

**DOI:** 10.1016/j.molimm.2007.06.144

**Published:** 2008-01

**Authors:** Natalie J. Hepburn, Jayne L. Chamberlain-Banoub, Anwen S. Williams, B. Paul Morgan, Claire L. Harris

**Affiliations:** a Department of Medical Biochemistry and Immunology, School of Medicine, Cardiff University, Heath Park, Cardiff CF14 4XN, United Kingdom; b Department of Rheumatology, School of Medicine, Cardiff University, Heath Park, Cardiff CF14 4XN, United Kingdom

**Keywords:** AChR, acetylcholine receptor, ADEAE, antibody-augmented demyelinating EAE, AP, alternative pathway, BuTx, Bungarotoxin, C, complement, CHO, Chinese hamster ovary, CP, classical pathway, CR1, complement receptor 1, CRegs, complement regulators, DAF, decay accelerating factor, EAE, experimental autoimmune encephalitis, EAMG, experimental autoimmune myasthenia gravis, FACS, fluorescent activated cell sorting, HRPO, horseradish peroxidase, MAC, membrane attack complex, NMJ, neuromuscular junction, OPD, 1,2-phenylenediamine dihydrochloride, PBS, phosphate-buffered saline, sCR1, soluble recombinant CR1, SCR, short-consensus repeat, sCrry, soluble recombinant form of rat Crry containing the four N-terminal SCRs, SPR, surface plasmon resonance, Complement, Crry, EAMG, Myasthenia gravis, Therapy

## Abstract

Despite its vital role in innate immunity, complement is involved in a number of inflammatory pathologies and has therefore become a therapeutic target. Most agents generated for anti-complement therapy have short half-lives in plasma, or have been of mouse or human origin, thereby limiting their use either to murine models of disease or to short-term therapy. Here we describe the generation of a long-acting rat therapeutic agent based on the rat complement inhibitor, Crry. Characterisation of various soluble forms of Crry demonstrated that the amino-terminal four short-consensus repeat domains were required for full regulatory and C3b-binding activities. Fusion of these domains to rat IgG2a Fc generated an effective complement inhibitor (rCrry-Ig) with a circulating half-life prolonged from 7 min for Crry alone to 53 h for rCrry-Ig. Systemic administration of rCrry-Ig over 5 weeks generated a weak immune response to the recombinant agent, however this was predominantly IgM in nature and did not neutralise Crry function or cause clearance of the agent from plasma. Administration of rCrry-Ig completely abrogated clinical disease in a rat model of myasthenia gravis whereas soluble Crry lacking the immunoglobulin Fc domain caused a partial response. rCrry-Ig not only ablated clinical disease, but also prevented C3 and C9 deposition at the neuromuscular junction and inhibited cellular infiltration at this site. The long half-life and low immunogenicity of this agent will be useful for therapy in chronic models of inflammatory disease in the rat.

## 1 Introduction

The complement (C) system forms part of the innate immune system; activation brings about target cell death or damage, opsonisation of pathogens, induction of inflammatory responses and clearance of immune complexes. The critical activation step is enzymatic cleavage of C3 or C4 by multi-molecular enzymes termed convertases, and deposition of the active components (C3b or C4b, respectively) on the target membrane. Nascent C3b and C4b bind indiscriminately to pathogens and adjacent host cells. To prevent inappropriate damage, self-cells express on their membranes complement regulatory proteins (CReg). These function by inactivating the convertases formed during C activation or by preventing formation of the membrane attack complex (MAC). In humans, membrane cofactor protein (MCP; CD46), decay accelerating factor (DAF; CD55) and complement receptor 1 (CR1; CD35) inactivate the convertases by accelerating their natural decay or by acting as a cofactor for the factor I (fI) mediated cleavage of C3b and C4b (Kim and Song, 2006). Rodents have a unique regulator of the convertases called Crry which both increases decay of the convertases as well as acting as a cofactor for fI (Wong and Fearon, 1985; Quigg et al., 1993; Takizawa et al., 1994; Li et al., 1993; Kim et al., 1995). All these CReg are comprised from short-consensus repeats (SCR), domains of approximately 60 amino acids in which the functional activity of the protein resides. CR1 consists of 30 SCR domains, DAF and MCP have four whilst rat Crry is comprised from either 6 or 7 SCRs due to alternative splicing of the gene.

In health, CReg are sufficient to protect the host from complement damage. However, in disease, dysregulated C activation can damage self-cells and exacerbate pathology. Complement is implicated in ischaemia/reperfusion injury, transplant rejection and a number of inflammatory diseases, such as systemic lupus erythematosus, autoimmune arthritis, multiple sclerosis and myasthenia gravis where activation triggers and sustains a ‘vicious cycle’ of inflammation and tissue damage (Morgan and Harris, 2003). In myasthenia gravis, an antibody-mediated disease of the neuromuscular junction (NMJ), C activation products have been found at the motor end plate (Lennon et al., 1978; Engel and Arahata, 1987; Sahashi et al., 1980). Involvement of C in pathology is demonstrated in rodent models by protective effects of C5 or C6 deficiency (Christadoss, 1988; Chamberlain-Banoub et al., 2006), amelioration of disease by anti-C therapeutics targeted either to activation pathways or to C5 (Piddlesden et al., 1996; Hepburn et al., 2007), and enhancement of disease in mice deficient in CReg (Morgan et al., 2006).

Due to the implication of C in inflammatory diseases a number of different anti-C therapeutics has been developed. Some of these reagents have reached clinical trials. A soluble, recombinant form of CR1, sCR1, has been tested in numerous animal models and reached clinical trials for acute conditions such as adult respiratory distress syndrome and cardiopulmonary bypass (Zimmerman et al., 2000; Rioux, 2001). Pexelizumab, a single chain Fv (scFv) which binds human C5 and prevents its cleavage, is in advanced stages of development for use in myocardial infarction (Whiss, 2002; Fleisig and Verrier, 2005). These reagents have been limited to acute therapies as they have short circulating half-lives necessitating constant administration in vivo. Eculizumab, an intact monoclonal antibody that binds C5, has a longer half-life in vivo and has been particularly successful in treatment of paroxysmal nocturnal haemoglobinuria and is being trialled in other chronic autoimmune indications (Hill et al., 2005; Kaplan, 2002).

A number of different approaches have been used to increase the circulating half-lives of therapeutic reagents. One approach has been generation of antibody-like molecules where the therapeutic moiety replaced the Fab arms of antibody. This approach was used to increase the circulating half-life of the C inhibitor, DAF, from 20 min to 33 h and to create a murine Crry-Ig with a half-life of 40 h (Harris et al., 2002; Quigg et al., 1998). Such reagents have proved therapeutically effective in antigen-induced arthritis, glomerulonephritis and intestinal ischaemia–reperfusion injury (Harris et al., 2002; Quigg et al., 1998; Rehrig et al., 2001). Reagents generated to date have been based upon human or mouse proteins, limiting their use in rat models due to their immunogenicity. In order to enable the testing of such reagents in rats we generated rCrry-Ig, a rat CReg-rat Fc protein consisting of rat Crry and the Fc of rat IgG2a. We compared rCrry-Ig to soluble Crry lacking an Fc domain both in vitro and in vivo. Tethering Crry to the Fc created a reagent with a greatly increased in vivo half-life that was capable of protecting against disease in a model of myasthenia gravis. This result paves the way for testing such reagents in chronic disease models in the rat.

## 2 Materials and methods

### 2.1 Materials

Chemicals and reagents were from Fisher Scientific (Loughborough, UK) or Sigma (Poole, UK) unless otherwise stated. All tissue culture reagents and plastics were from Invitrogen Life Technologies (Paisley, UK). pDR2ΔEF1α was a gift from Dr. I. Anegon (INSERM U437, Nantes, France; Charreau et al., 1994). The cell line expressing the monoclonal antibody, TLD1C11, was from Prof. W. Hickie (Dartmouth, USA). Chinese hamster ovary (CHO) cells expressing surface-bound rat Crry were from Dr. O.B. Spiller (Cardiff, UK). Sheep erythrocytes in Alsever's solution were from TCS microbiology (Claydon, UK), guinea pig erythrocytes and rat serum were from the local animal facility. Rabbit anti-rat Crry polyclonal antiserum and polyclonal sheep anti-human C9 were prepared in-house using standard immunisation procedures. The hybridoma expressing mouse anti-human C3b (C3-30) was a gift from Novartis (Horsham, UK; Kemp et al., 1992). Polyclonal goat anti-rat C3c was from Nordic Laboratories (Copenhagen, Denmark) and polyclonal sheep anti-human C3c antiserum was obtained from The Binding Site (Birmingham, UK). The rat hybridoma TIB-175, secreting the anti-AChR mAB35 was obtained from the American Tissue Culture Collection; antibody was purified as described previously (Tzartos et al., 1987; Chamberlain-Banoub et al., 2006). Mouse anti-rat CD68 was obtained from Serotech (Oxford, UK).

PBS is 8.1 mM Na_2_PO_4_, 1.5 mM KH_2_PO_4_, 137 mM NaCl, 2.7 mM KCl, pH 7.4. CFD (Complement fixation diluent; Oxoid) is 2.8 mM barbituric acid, 145.5 mM NaCl, 0.8 mM MgCl_2_, 0.3 mM CaCl_2_, 0.9 mM sodium barbital, pH 7.4. Alternative pathway (AP) buffer is 5 mM sodium barbitone, 150 mM NaCl, 7 mM MgCl_2_, 10 mM EGTA, pH 7.4. FACS buffer is PBS containing 0.1% sodium azide, 1% BSA and 10 mM EDTA. Borate-buffered saline is 100 mM boric acid, 25 mM sodium borate, 75 mM NaCl, pH 8.3.

### 2.2 Generation of cell lines expressing recombinant proteins

DNA encoding the signal peptide and the three (C-terminal residue Leu193) or four (C-terminal residue Lys256) N-terminal SCR of rat Crry was amplified by PCR from rat oligodendroglioma cell cDNA (33B; obtained from ECACC), the antisense primer encoded a stop codon. DNA was ligated into the pDR2ΔEF1α expression vector. To generate the rCrry-Ig protein, DNA encoding the Fc (hinge, CH2 and CH3 domain) of rat IgG2a was ligated into pDR2ΔEF1α to generate the pDR2-Fc vector and DNA encoding the signal peptide and four SCR of rat Crry (without stop codon) was cloned into the vector upstream and in frame with the DNA encoding the Fc. Vent proof reading polymerase (NEB, Hitchin, UK) was used for the PCR and sequencing confirmed that no errors had been introduced. CHO cells were transfected with the vectors using LipofectAMINE (Invitrogen Life Technologies) according to the manufacturer's instructions. Stable lines were established by selection with 400 μg/ml Hygromycin B.

### 2.3 Protein purification

Supernatant was collected from transfected cells and purified by affinity chromatography on monoclonal anti-Crry. This column was generated by coupling 100 mg TLD1C11 (anti-Crry) to CNBr-activated Sepharose (GE Healthcare, St. Albans, UK) according to the manufacturer's instructions. Supernatant was passed over the column, the column washed with PBS and the recombinant proteins eluted using 0.1 M glycine/HCl pH 2.5. Eluted protein was neutralised with 1 M Tris, dialysed into PBS and concentrated by ultrafiltration.

### 2.4 SDS-PAGE and Western blot analysis

Purified proteins were subjected to SDS-PAGE and separated proteins were stained with Coomassie R-250. For Western blot analysis, proteins were transferred to nitrocellulose (VWR, Lutterworth, UK), blocked with 5% (w/v) non-fat milk in PBS and incubated with the relevant antibodies. To detect rCrry-Ig the membrane was incubated with donkey anti-rat IgG HRPO (Jackson ImmunoResearch, Luton, UK) at 1/10,000 in 5% PBS milk. To detect sCrry proteins, the membrane was incubated with TLD1C11 at 1 μg/ml in 5% PBS milk followed by donkey anti-mouse IgG HRPO (Horseradish peroxidase; Jackson ImmunoResearch) at 1/10,000 in 5% PBS milk. To detect C3 fragments the membrane was incubated with polyclonal anti-human C3c antiserum (cross-reactive with rat C3) at 1/1000 followed by anti-sheep Ig HRPO (Binding site, Birmingham, UK) at 1/1000. Between primary and secondary antibody incubations and prior to development membranes were washed three times in PBS/0.1% Tween-20 followed by three washes in PBS. Bands were visualised using ECL (GE Healthcare) and autoradiographic film.

### 2.5 SPR analysis

The surface plasmon resonance (SPR) analysis was carried out on a Biacore 3000 (Biacore International, Uppsala, Sweden) following previously reported methods (Harris et al., 2007). In brief, a small nidus of human C3b (50RU) was amine-coupled to a chip surface, human factors B and D were flowed to form a C3 convertase which in turn was used to deposit rat C3b (1000RU) on the chip surface. Rat C3b was covalently coupled to the surface by nucleophilic attack on the internal thioester, giving native orientation. Human C3b does not interact with rat Crry, therefore there is no interference in the Crry-rat C3b interaction (our unpublished observation). Different concentrations of the sCrry proteins were separately flowed over the chip surface and the dissociation equilibrium constant (*K*_D_) calculated from steady state kinetics.

### 2.6 Haemolysis assays

Classical pathway (CP) haemolysis assays were carried out as described previously (Harris et al., 2002). In brief, antibody-coated sheep erythrocytes were incubated with a concentration of serum titrated to give 60% lysis and different concentrations of the test protein. Similarly, in the AP haemolysis assay, 1.5% guinea pig erythrocytes were incubated with a concentration of rat serum titrated to give 60% lysis and different concentrations of the test protein; the AP assay was carried out in AP buffer. Lysis was calculated by release of haemoglobin from erythrocytes.

The in vivo complement inhibitory activity of the proteins was determined following a previously reported method (Hepburn et al., 2007). In brief, antibody-coated sheep erythrocytes were incubated with differing concentrations of pre-treatment serum or with serum from rats administered the recombinant proteins. For each time point after reagent administration, the concentration required to give 50% lysis was calculated and expressed relative to the concentration of pre-treatment serum required to give the same degree of lysis.

### 2.7 Cofactor assay

The ability of the recombinant forms of Crry to act as a cofactor for the fI-mediated cleavage of C3b-like C3 (C3_MA_) was investigated following a previously reported method (Fraser et al., 2002). C3 was incubated with methylamine in borate-buffered saline pH 8.3 to form C3_MA_. C3_MA_ (2.5 μg) was incubated with fI (0.5 μg) and a test Crry protein (2 μg) for 4 h at 37 °C. Cleavage by fI was detected by resolving the incubations on a reducing SDS-PAGE gel and Western blotting. Cleavage of the α-chain was detected by probing the blot with polyclonal anti-human C3c antiserum.

### 2.8 In vivo half-life

The in vivo half-lives of the recombinant Crry proteins were established as described previously (Harris et al., 2002). Five female Lewis rats (Charles River, average weight 180 g) were administered ^125^I labelled rCrry-Ig or sCrry intravenously. Plasma was removed at specific times following administration, protein was precipitated and protein-bound ^125^I calculated. Clearance was calculated according to the method of Fraker and Speck (1978).

### 2.9 Immune response to therapeutic reagent

Female Wistar rats (five per group, average weight 180 g; Charles River) were injected with 1 mg/kg rCrry-Ig on days 0, 3, 7, 14, 21 and 28 through the tail vein. Blood samples were collected via the tail vein at specific time points and serum isolated. An ELISA was used to detect the generation of anti-rCrry-Ig antibodies. The ELISA plate was coated with rCrry-Ig, incubated with test serum (diluted 1/50) from each time point and rCrry-Ig specific antibodies were detected using HRPO-conjugated antibodies against rat IgM, IgG1, IgG2b or IgG2c (Southern Biotech, Birmingham, AL, USA). The ELISA was developed using OPD and the reaction stopped with 10% sulphuric acid. The presence of rCrry-Ig in the serum was detected using an ELISA plate coated with TLD1C11 (mouse anti-rat Crry), incubated with serum or standard concentrations of rCrry-Ig and probed with anti-rat IgG HRPO (Jackson ImmunoResearch). rCrry-Ig present in serum was quantified from the standard curve.

To establish whether the generated antibodies were function-blocking, CHO cells expressing transmembrane rat Crry (50 μl at 2 × 10^6^ ml^−1^) were incubated with serum (10%, v/v) from test animals or rabbit polyclonal anti-Crry antiserum (10%, v/v) for 1 h at 4 °C. Cells were also incubated with non-immune rabbit or rat serum as negative controls, all sera were heat inactivated. Following three washes in FACS buffer, the cells were incubated at 37 °C for 30 min in 2% (v/v) rat serum. Cells were washed and stained with mouse anti-human C3b (C3-30; cross-reactive with rat C3b) for 30 min at 4 °C followed by an incubation with donkey anti-mouse Ig RPE (Dako) for 1 h at 4 °C. The cells were washed, resuspended and analysed on a Becton Dickinson FACSCalibur.

### 2.10 In vivo complement inhibitory function

Female Lewis rats (five per group, average weight 180 g; Charles River) were injected intravenously with rCrry-Ig at 20 mg/kg or sCrry at 10 mg/kg. Blood was collected from the tail vein at specific time points, serum was isolated and processed as described in Section 2.6 above.

### 2.11 Experimental autoimmune myasthenia gravis (EAMG)

EAMG was induced in female Lewis rats (Charles River; average weight 180 g) as described previously (Chamberlain-Banoub et al., 2006; Piddlesden et al., 1996). In brief, disease was induced on day 0 using 1 mg/kg mAb35 in PBS (i.p.). mAb35, produced against *Electrophorus electricus* electric organ muscle-type nicotinic AChR, binds the alpha subunit of the AChR and cross-reacts with rat AChR. At the same time as disease induction, animals were given PBS (1 ml), rCrry-Ig (20 mg/kg) or sCrry (10 mg/kg; equimolar amount of Crry) through the tail vein (six animals per group). Weight change and clinical score were monitored; increasing clinical score was indicative of muscle weakness and paralysis. At 52 h post-induction, all the PBS group were sacrificed due to disease severity along with one animal from each of the sCrry and rCrry-Ig groups for comparative histology, the remaining animals were sacrificed at 183 h post-induction. The soleus muscles were isolated from the sacrificed animals, flash frozen and cut into 10 μm sections. The sections were processed and stained for C3, C9 and inflammatory cell infiltrate as described previously (Hepburn et al., 2007).

## 3 Results

### 3.1 Generation of soluble Crry proteins

**Fig. 1:**
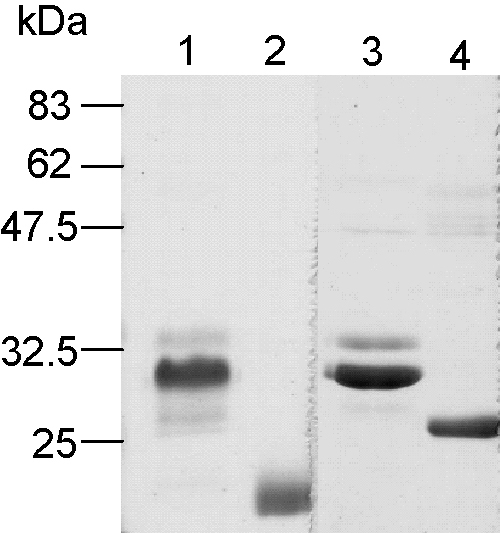
Analysis of purified sCrry reagents by SDS-PAGE. The proteins were purified from cell culture supernatant by anti-Crry affinity chromatography and subjected to SDS-PAGE (11% gel). Lanes 1 and 3, sCrry non-reduced and reduced, respectively. Lanes 2 and 4, sCrry (3SCRs) non-reduced and reduced, respectively.

To generate the soluble Crry proteins, DNA encoding the three or four N-terminal SCR domains of rat Crry was ligated into the pDR2ΔEF1α vector. CHO cells were transfected with the plasmids and stable cell lines were generated. Supernatant was harvested and proteins were purified using a monoclonal anti-Crry affinity column. Purified proteins were analysed by SDS-PAGE (Fig. 1). The molecular weight of sCrry was 28 kDa under reduced and non-reduced conditions. The molecular weight of sCrry (3SCRs) was 21 kDa under non-reducing conditions and 25 kDa under reducing conditions, an increase in weight characteristic of SCR-containing proteins. The molecular weights were corroborated by mass spectrometry (sCrry 28.248 Da; sCrry (3SCRs) 21.216 Da; data not shown).

### 3.2 Definition of the minimal functional unit of Crry

**Fig. 2:**
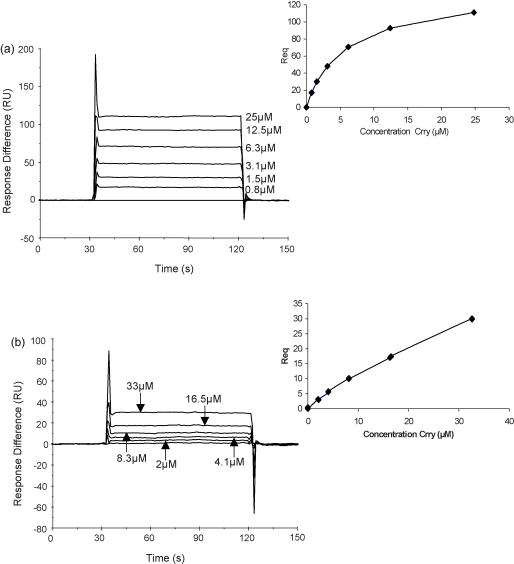
SPR analysis of the interaction of the sCrry reagents with rat C3b. Rat C3b was coupled covalently to the chip surface using the internal thioester bond and the interaction with sCrry (a) and sCrry (3SCRs) (b) was analysed. The affinity of the interaction was analysed by steady state kinetics (see inset).

To identify the minimal functional unit of Crry, the binding of sCrry and sCrry (3SCRs) to rat C3b was compared using SPR. Rat C3b was coupled to the chip surface and different concentrations of each protein flowed across the surface. The binding of sCrry (3SCRs) to C3b was dramatically reduced compared to sCrry (Fig. 2a and b). The affinity of the two proteins for C3b was calculated using steady state analysis (insets Fig. 2a and b). The dissociation equilibrium constant (*K*_D_) for sCrry protein interaction with C3b was 5.1 × 10^−6^ M (*χ*^2^ = 0.4) whereas that for the sCrry (3SCRs) protein was too low to calculate accurately; there was minimal binding even at 33 μM. The sCrry (3SCRs) protein also demonstrated weak inhibitory activity in AP haemolysis assays (IH50 = 545 nM ± 51; sCrry IH50 = 74.1 nM ± 1.8; data not shown).

### 3.3 Inhibitory function of rCrry-Ig

**Fig. 3:**
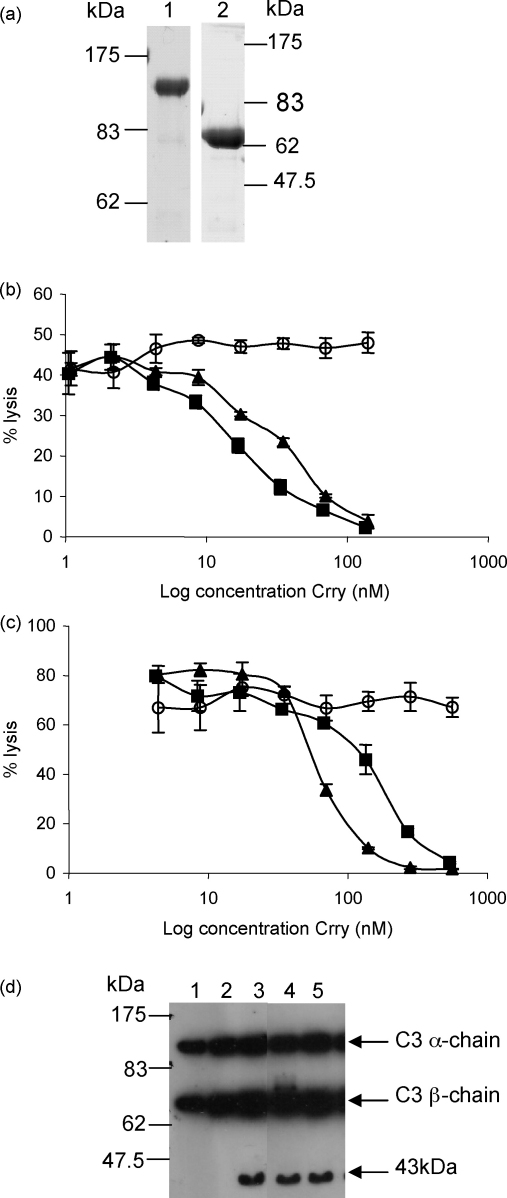
Inhibition of C by rCrry-Ig and sCrry. (a) rCrry-Ig was purified from cell culture supernatant by anti-Crry affinity chromatography and analysed by SDS-PAGE under non-reducing (7.5% gel; lane 1) or reducing conditions (10% gel; lane 2). (b) Antibody-sensitised erythrocytes were attacked with rat C and the ability of rCrry-Ig (■) and sCrry (▴) to inhibit CP-mediated haemolysis was compared with non-regulatory rat Ig (○). (c) Guinea pig erythrocytes were incubated with rat serum and the ability of rCrry-Ig (■), sCrry (▴) and rat Ig (○) to prevent AP-mediated haemolysis was determined. Each data point represents the mean value ±1S.D. of three determinations. (d) Methylamine inactivated C3 (C3_MA_) was incubated with fI in the presence of a cofactor: sCR1 (lane 3), rCrry-Ig (lane 4) or sCrry (lane 5). Ability of the reagent to act as cofactor was determined by SDS-PAGE on a 10% reduced gel followed by Western blotting. The blot was probed with anti-human C3c (cross-reactive with rat C3) followed by HRPO-conjugated anti-sheep Ig. Controls included C3_MA_ only (lane 1) and C3_MA_ with factor I (lane 2).

Given that the four N-terminal SCRs of rat Crry were identified as the minimal functional unit of Crry, a rCrry-Ig fusion protein was generated and purified as described for the sCrry reagents (Fig. 3a). The Fc domain was added to prolong the in vivo half-life as that of 4SCR was predicted to be short (Harris et al., 2002). The rCrry-Ig fusion protein had a molecular weight of 140 kDa by non-reducing SDS-PAGE and 70 kDa when the disulphide bonds at the hinge region were reduced (Fig. 3a; lane 2).

The abilities of rCrry-Ig and sCrry to inhibit C in vitro and the ability of the proteins to act as cofactors for the fI-mediated cleavage of C3b were compared. Both reagents inhibited the CP (Fig. 3b). The proteins were compared in terms of Crry molarity as approximately half the mass of rCrry-Ig is comprised of the Fc. This comparison revealed that rCrry-Ig was more potent than sCrry at inhibiting the CP (IH50 rCrry-Ig = 16.7 ± 0.6 nM; IH50 sCrry = 31.0 ± 0.7 nM). In the AP, sCrry was more effective at inhibiting lysis (Fig. 2c; rCrry-Ig IH50 = 167.8 ± 17.8 nM; sCrry IH50 = 74.1 ± 1.8 nM, *p* < 0.05).

In the cofactor assay, both proteins supported production of a 43 kDa fragment from rat C3_MA_ demonstrating that rCrry-Ig and sCrry are effective cofactors for the fI-mediated cleavage of C3b (Fig. 3d). Incubation with the positive control, sCR1, also produced this fragment.

### 3.4 Circulating half-life of rCrry-Ig

**Fig. 4:**
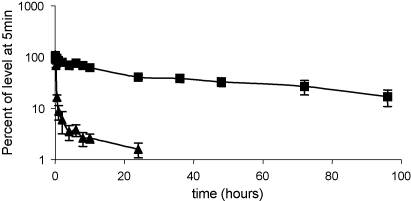
In vivo half-life of rCrry-Ig and sCrry. Radiolabelled rCrry-Ig (■) and the sCrry reagent (▴) were administered to rats intravenously, blood was removed at specific time points and protein bound counts determined. This was expressed as a percent of protein bound counts present at 5 min post-administration and the beta phase half-life determined (Waller, 1994). Each data point represents the mean of five animals ±1S.D.

To assess the effect the Fc had on the in vivo clearance of Crry, rCrry-Ig and sCrry were radiolabelled with ^125^I. A single intravenous dose of either reagent was administered to rats and samples of blood were removed at intervals and protein bound counts were measured. sCrry was rapidly cleared from the circulation with a half-life of 7 min. rCrry-Ig had a significantly prolonged half-life of 53 h (*p* < 0.001) (Fig. 4).

### 3.5 Generation of an immune response to rCrry-Ig

**Fig. 5:**
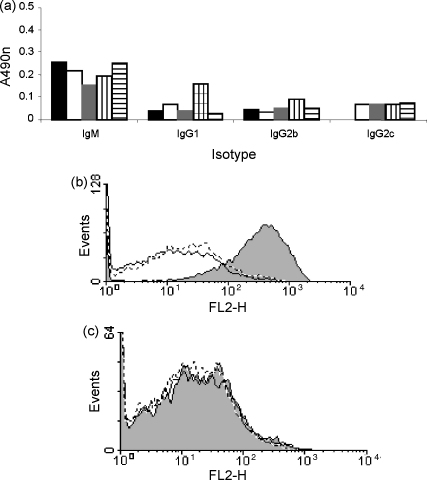
Immunogenicity of rCrry-Ig. Rats were administered rCrry-Ig at days 0, 3, 7 and weekly thereafter, and blood was collected at certain time points. The presence of different antibodies against rCrry-Ig was detected by ELISA in the day 35 serum (a). Each bar represents a different animal. (b) Rabbit anti-Crry antiserum (shaded; positive control; mean fluorescence 399.6), buffer (solid line; mean fluorescence 51.3) or normal rabbit serum (dashed line; mean fluorescence 38.5) was incubated with CHO cells expressing rat Crry prior to incubation with active rat C. Deposition of C3b on the CHO cells was detected by flow cytometry. (c) Serum from an immune animal (shaded; mean fluorescence 41.72 ± 7.09), buffer (solid line; mean fluorescence 30.67) or normal rat serum (dashed line; mean fluorescence 38.5) was incubated with the CHO-Crry cells. The cells were attacked with C and C3b deposition detected as above. No increase in C3b deposition was observed following incubation with the immune sera. This histogram is representative of results from five different animals.

We investigated whether generation of a Crry/antibody Fc chimeric protein created an immunogenic neoepitope at the join between the two proteins. Animals were administered rCrry-Ig intravenously on days 0, 3, 7 and weekly thereafter to 28 days. Blood was collected at weekly intervals, serum harvested and production of antibodies was assessed by ELISA. A weak immune response was detected to rCrry-Ig (Fig. 5a). All animals produced an IgM response in early bleeds (from day 7) with subsequent class switching to IgG1, IgG2b and IgG2c from day 21. The IgG2a response could not be assessed in this experiment as the ELISA coat contained the Fc of IgG2a resulting in cross-reactivity. Coating an ELISA with rCrry-Ig, sCrry and a control fusion protein with the same Fc revealed that the immune response had been mounted against all domains in the chimeric molecule and not just the join between Crry and the Fc domain (data not shown).

The ability of these antibodies to inhibit the function of rCrry-Ig was investigated using CHO cells expressing surface-bound Crry. Incubation of CHO cells with test sera resulted in antibody binding (data not shown), deposition of C3 on the cell surface was assessed following incubation with rat serum as a source of C. Incubation with polyclonal anti-Crry as a positive control resulted in enhanced C3 deposition compared to buffer or serum only (Fig. 5b), whereas pre-incubation with test sera did not enhance C3 deposition demonstrating the non-function blocking nature of the antibodies (Fig. 5c). In support of this, quantification by ELISA of rCrry-Ig remaining in the circulation throughout 5 weeks of repeated administration demonstrated steady state levels of reagent without evidence of enhanced clearance (Table 1).

### 3.6 C inhibition in vivo

**Fig. 6:**
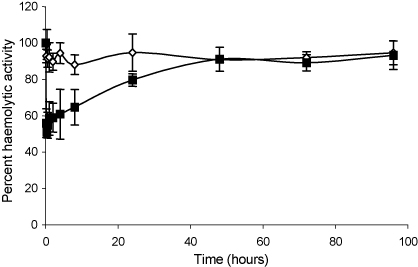
Effect of rCrry-Ig on rat C activity in vivo. Rats were administered rCrry-Ig (■) or sCrry (◊) and blood was collected at specific time intervals. The serum was isolated and used as a source of C to lyse antibody-coated erythrocytes. The lytic ability of the serum was compared to that of a pre-bleed. Each data point represents the mean of five animals ±1S.D.

A single dose of the proteins was administered intravenously to normal rats in order to assess their C inhibitory ability. Serum was harvested at specific time intervals and haemolytic activity was assessed. Administration of sCrry had no discernable effect on serum haemolytic activity even at the early time points, likely due to rapid clearance, whereas animals administered rCrry-Ig had reduced serum haemolytic activity when compared to a pre-bleed, 40–50% for the first 8 h after administration (*p* < 0.05; Fig. 6). Reduction of serum haemolytic activity was detected for 24 h post-administration. It should be noted that this assay, because it involves dilution of test serum, underestimates the true inhibitory effect of the agent, which is highly concentration dependent.

### 3.7 rCrry-Ig protection against EAMG

**Fig. 7:**
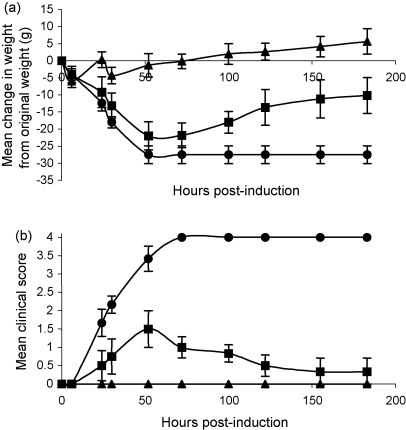
Clinical assessment of rats with EAMG. EAMG was induced in rats and weight change (a) and clinical score (b) were monitored. Control animals (●) rapidly developed a clinical score and lost weight, these animals were sacrificed at 52 h and thereafter their score was scored 4. rCrry-Ig-treated animals (▴) were protected from disease whilst sCrry-treated animals (■) developed slight clinical disease and then recovered.

**Fig. 8:**
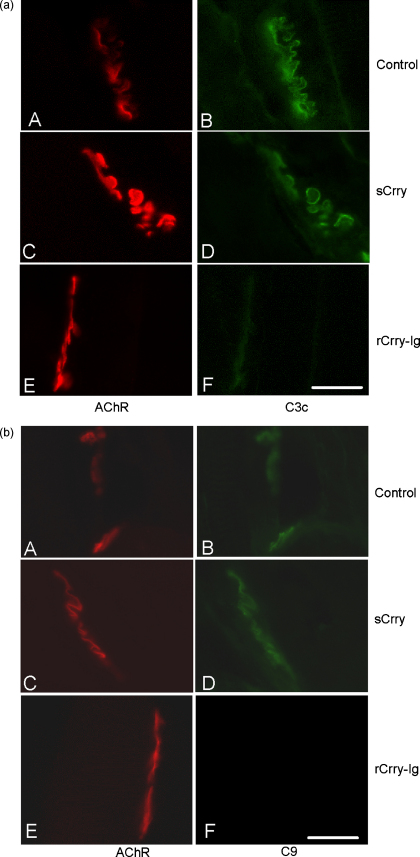
C deposition at the NMJ. (a) The soleus muscle isolated from the control group, sCrry- and rCrry-Ig-treated animals at 52 h was sectioned and stained for AChR using BuTx (left-hand column) and C3 (right-hand column). (b) Similarly, the isolated soleus muscle was sectioned and stained for AChR using BuTx (left-hand column) and C9 (right-hand column). Bar represents 400 μm, representative images are shown.

The therapeutic effect of rCrry-Ig and sCrry was assessed in a rat model of EAMG. Untreated animals rapidly developed severe weight loss and paralysis and had to be sacrificed by 52 h. Therapy with rCrry-Ig prevented weight loss (Fig. 7a) and totally ablated clinical disease (Fig. 7b). Animals treated with sCrry had mild disease with weight loss and an increasing clinical score until 52 h post-induction when they began to recover; clinical disease was always less severe than in control animals. Immunohistochemical analysis at the end plate demonstrated significantly reduced C3 and C9 deposition in the rCrry-Ig animals compared to the control and sCrry animals (Fig. 8; Table 2). Immunohistochemical analysis and H&E staining demonstrated reduced levels of cellular infiltration in the rCrry-Ig-treated animals (Table 2).

## 4 Discussion

The demonstration of an involvement of C in various inflammatory pathologies has been the driving force behind the generation of recombinant biologicals designed to inhibit C in disease. Amongst these reagents are soluble recombinant CReg, including sCR1. This reagent has been protective against tissue injury in several animal models including EAMG, antibody-augmented demyelinating experimental allergic encephalomyelitis (ADEAE), antigen-induced arthritis (AIA), glomerulonephritis and ischaemia/reperfusion injuries (Piddlesden et al., 1994, 1996; Goodfellow et al., 1997; Weisman et al., 1990). However, the use of soluble recombinant CRegs has been limited by rapid clearance in vivo resulting in the need for repeated administration, rendering them unsuitable for long-term therapy. A number of different approaches have been employed to increase the half-lives of such reagents. These include addition of an albumin binding site to CR1, targeting of CReg to membranes or the site of C activation and generation of CReg-Ig fusion proteins (Makrides et al., 1996; Smith and Smith, 2001; Song et al., 2003; Atkinson et al., 2005).

Fusion of proteins to Ig domains has been used widely to increase the half-life of many therapeutic proteins, including CTLA-4 and TNF-α receptor (Linsley et al., 1992; Pugsley, 2001). We have previously described CReg-Ig fusion proteins where the rat CReg, DAF or CD59, was fused to a human immunoglobulin Fc domain (Harris et al., 2002). DAF-Ig had prolonged circulating half-life and was effective in a rat model of AIA; we could not test this reagent in models of chronic disease due to immunogenicity of the human Fc domain. Others have generated an Fc fusion protein comprising mouse Crry and murine IgG1 Fc, this has also proven efficacious in many murine models of disease (Quigg et al., 1998; Rehrig et al., 2001; Leinhase et al., 2006). We describe here the generation of a reagent comprising rat Crry fused to rat IgG2a Fc; as both domains are derived from rat proteins this agent should have low immunogenicity and be suitable for therapy in both acute and chronic models of inflammatory disease in the rat.

Crry contains both decay accelerating and cofactor activities and is a powerful regulator of C (Li et al., 1993; Kim et al., 1995). Therapeutic molecules combining both activities in one molecule have proven to be more effective than those comprising one activity alone (Christiansen et al., 1996; Higgins et al., 1997). We first defined the domains of rat Crry necessary for regulatory function; our previous findings have shown that the 4 N-terminal SCRs were capable of regulating the CP and act as a cofactor for the fI mediated cleavage of C3b (Fraser et al., 2002). Recombinant Crry constructs were generated that comprised either the first three or four N-terminal SCR of Crry (Fig. 1), these were compared for their ability to bind the ligand, rat C3b. The affinity of sCrry for C3b was 5.1 μM, deletion of the fourth SCR markedly decreased ability of the reagent to bind C3b, revealing that the fourth N-terminal SCR contains an important C3b binding site (Fig. 2). We therefore generated a construct comprising the N-terminal 4SCR fused to rat IgG2a Fc and tested its ability to inhibit C in vitro (Fig. 3). Both rCrry-Ig and sCrry inhibited in a CP haemolysis assay, rCrry-Ig showed improved function presumably due to the ability of the two Crry moieties in the protein to act synergistically. A similar effect has been noted previously with CR1 where multiple binding/functional sites enhanced inhibition of the C3 and C5 convertases (Krych-Goldberg et al., 1999, 2005). In the case of CR1, increased binding avidity may explain enhanced regulation of the C3 convertase, whereas ability to bind to both C3b/C4b subunits within the enzyme may explain enhanced regulation of the C5 convertase where exact spacing between the active sites is crucial for efficient decay. Either mechanism could be responsible for enhanced function of Crry-Ig in the CP that we describe here. Inhibition of C by rCrry-Ig in an AP haemolysis assay was slightly less efficient when compared to the same reagent lacking an Fc domain. We have demonstrated that SCR4 of Crry is required to bind C3b (Fig. 2), location of this domain close to the antibody hinge and Fc may therefore restrict access to the ligand. This effect has been noted previously with DAF-Ig where steric hindrance imparted by tethering DAF to the Fc reduced function two-fold (Harris et al., 2002).

Tethering the four N-terminal SCRs of rat Crry to an Fc dramatically increased the circulating half-life as expected (Fig. 4), an effect of increasing size of the protein (limiting filtration by the kidney) and interaction with Fc receptors which serve to actively retain the protein in the circulation (Ghetie and Ward, 1997; Ghetie and Ward, 2000). Long-term administration of rCrry-Ig resulted in a weak immune response, although the antibodies generated were predominantly IgM and did not result in clearance of the reagent or inhibit its function (Fig. 5). Similar immune responses have been noted to other therapeutics, but this has not limited their use in the clinic. The immune response may have been caused by different post-translational modifications carried out by the CHO cells used to express the reagent in vitro. Others have shown that high concentration of ammonium ions, produced by cellular metabolism, alters glycosylation in CHO cells (Chen and Harcum, 2005). Recombinant follicle stimulating hormone (FSH) expressed in CHO cells differed from native FSH in terms of sialyation of carbohydrates (Recombinant Human FSH Product Development Group, 1998). Selection of an alternative cell line for expression of the recombinant therapeutic may ablate the observed immunogenicity.

At the doses administered in our experiments, animals treated with rCrry-Ig had reduced serum C activity (50% when compared to pre-bleed; Fig. 6), this was sufficient to prevent any clinical score in rat EAMG (Fig. 7). Treatment with rCrry-Ig was so effective that no C3 or C9 deposition was evident at the NMJ (Fig. 8). Despite its short half-life, sCrry reduced clinical score, although this was not as dramatic as therapy with rCrry-Ig. As EAMG is an acute disease, the presence of sCrry during the initial C activation may have reduced C amplification and therefore lessened the degree of injury. We have previously shown that an acute phase response early in the course of this disease increases plasma complement activity (Hepburn et al., 2007), the presence of sCrry at initial stages may dampen the acute response with therapeutic benefit. Both C3b and C9 deposition were detectable at the NMJ in the sCrry-treated group, showing that the reagent did not completely halt C activation suggesting it had a role in regulating the initial injury during disease establishment. The C3-staining evident in Fig. 8 may represent iC3b generated through the cofactor activity of sCrry, again accounting for some therapeutic benefit in the acute phase of the disease. The degree of cellular infiltration differed between the two groups, rCrry-Ig-treated animals had virtually no cellular infiltration, unlike the sCrry-treated animals. This reduction in cellular infiltration again confirms the efficacy of rCrry-Ig in regulation of C and reduction in levels of the anaphylatoxins C3a and C5a as well as MAC, all of which recruit inflammatory cells.

In conclusion, we have generated and characterised both in vitro and in vivo a long-lived and powerful inhibitor of rat C, rCrry-Ig. This reagent totally ablated clinical score in a rat model of acute inflammatory disease, EAMG, and inhibited C activation at the site of tissue damage (NMJ). rCrry-Ig had low immunogenicity when given long-term, thereby facilitating its use in rodent models of chronic inflammation, such as collagen-induced arthritis, and enabling the efficacy and side-effects of prolonged therapy in treatment of chronic inflammatory diseases to be assessed.

## Figures and Tables

**Table 1:** Serum concentrations of rCrry-Ig during the immune response study

Day	7	14	21	28	35
Concentration (μg/ml)	10.4	7.3	4.3	4.6	6.8

The concentration of rCrry-Ig, assessed by ELISA, in the serum isolated from one representative animal on certain days during the immune response study.

**Table 2:** Histological analysis of the NMJ in soleus muscle from EAMG animals

Group	C3 deposition	C9 deposition	Infiltration (H&E)	Infiltration (ED1 staining)
Control (52 h)				
1	++	+	2	2–3
2	++	−	2	2–3
3	++	+	3	3
4	+	+	3	2
5	+	+	3	2–3

sCrry-treated (183 h)				
1	++	++	1	2–3
2	±	+	2	2–3
3	++	++	3	2
4	+	++	3	3
5	++	+	3	3
52 h animal	++	++	1	1–2

rCrry-Ig-treated (183 h)				
1	−	−	1	1
2	±	−	1	0
3	+	−	1	0
4	−	−	1	0
5	±	−	1	0
52 h animal	±	−	0	0–1

Sections were double-stained for AChR (rhodamine-conjugated α-bungarotoxin) and either activated C3 or C9. Deposition of C3 and C9 were scored as follows: −, negative; ±, trace; +, weak; ++, strong. Infiltration was assessed following H&E staining and infiltrating macrophages were detected by immunofluorescence using ED1 (anti-CD68). Infiltration was scored as follows: 0, no infiltration; 1, up to 20% of the whole muscle infiltrated; 2, up to 40% of whole muscle infiltrated; 3, up to 60% of whole muscle infiltrated; 4, over 60% of muscle infiltrated. PBS control group were sacrificed at 52 h due to disease severity along with one animal from the two treatment groups (52 h animal). All other animals were sacrificed at 183 h.
